# Preliminary study on the feasibility of united compressed sensing with radial acquisition as a routine method for liver dynamic contrast-enhanced examination in elderly patients with malignancy

**DOI:** 10.1186/s13244-025-01936-4

**Published:** 2025-03-22

**Authors:** Heping Deng, Xiaolei Dong, Yu Zhang, Peng Zhou, Yakun He, Liu Yang

**Affiliations:** 1 Radiology Department, Sichuan Clinical Research Center for Cancer, Sichuan Cancer Hospital & Institute, Sichuan Cancer Center, Affiliated Cancer Hospital of University of Electronic Science and Technology of China, Chengdu, China; 2 Out-patient Department, Sichuan Clinical Research Center for Cancer, Sichuan Cancer Hospital & Institute, Sichuan Cancer Center, Affiliated Cancer Hospital of University of Electronic Science and Technology of China, Chengdu, China

**Keywords:** Magnetic resonance imaging, Dynamic contrast enhanced, Signal-to-noise ratio, Contrast-to-noise ratio, Qualitative analysis

## Abstract

**Objective:**

To explore the value of the united imaging compressed sensing with radial acquisition (uCSR) in liver dynamic contrast-enhanced examinations for elderly patients with malignancy.

**Methods:**

Hundred patients aged 65 years or over were randomly divided into two groups: 50 patients underwent liver dynamic contrast-enhanced scanning using the uCSR sequence during free breathing, and 50 patients underwent scanning using the three-dimensional volume interpolated breath-hold examination (3D-VIBE) sequence while holding breath. Two radiologists independently and subjectively evaluated the overall image quality and image artifacts with a five-point scale. Concurrently, two technologists measured the signal-to-noise ratio (SNR) and contrast-to-noise ratio (CNR) of the arterial, portal venous and delay phase images in both groups.

**Results:**

uCSR has superior overall image-quality and image-artifact scores (*z* = 2.342, *p* = 0.019; *z* = 2.105, *p* = 0.035). The 3D-VIBE images of the arterial phase have higher SNR than uCSR (*t* = 4.988, *p* = 0.000), with no significant difference in the CNR (*z* = 0.676, *p* = 0.499). In the portal venous phase, the SNR and CNR of the 3D-VIBE images are superior to those of uCSR (*z* = 5.674, *p* = 0.000; *t* = 3.638, *p* = 0.000). In the delay phase, the SNR of the 3D-VIBE is slightly better than the uCSR (*t* = 5.471, *p* = 0.000), and the CNR shows no significant difference (*z* = 1.258, *p* = 0.208).

**Conclusion:**

uCSR can be used as a method for liver dynamic contrast-enhanced scans in elderly patients with malignancy. It can improve patient comfort and reduce the failure rate of scans.

**Critical relevance statement:**

Our findings suggested that uCSR can be used for liver dynamic contrast-enhanced scans in elderly patients with malignancy, this preliminary study provided basis for it.

**Key Points:**

- The uCSR can suppress the impact of respiratory motion artifacts on images.
- The UCSR can perform dynamic enhanced scanning of the liver under free breathing dynamics.
- The uCSR is suitable for dynamic contrast-enhanced MR imaging of the liver in elderly patients with malignancy.

**Graphical Abstract:**

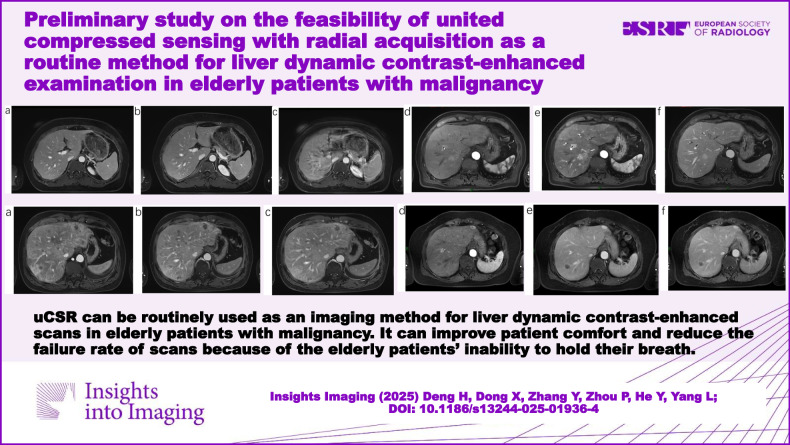

## Introduction

Population aging in China is accelerating, and the proportion of individuals aged ≥ 65 years is increasing [1]. Thus, the proportion of elderly patients who undergo liver magnetic resonance imaging (MRI) examinations is increasing. Because the liver is a dual blood supply organ, a three-phase scan (i.e., arterial, portal vein, and delayed phases) is particularly important. Routine liver dynamic contrast-enhanced scanning uses a sequence called three-dimensional volume interpolated breath-hold examination (3D-VIBE) [2]. This technique adopts the linear K-space filling method, which requires a high level of patient cooperation because scanning is performed during breath-holding. Each breath-holding period lasted approximately 15–20 s, and the number of breath-holding cycles ranged between 4 to 7 times. However, elderly patients, particularly those with malignancy, cannot perform breath-holding for 3D-VIBE. Thus, motion artifacts appear. Consequently, respiratory motion artifacts may appear in the images and render these images inadequate for clinical diagnosis [3]. Current imaging techniques can ensure dynamic contrast-enhanced liver scanning in a free-breathing state, including extra-dimensional golden-angle radial sparse parallel (XD-GRASP) with additional dimensions [4], Cartesian sampling, extra-dimensional VIBE (XD-VIBE) technology [5] and United Imaging compressed sensing with radial acquisition (uCSR). The uCSR technique combines compressed sensing technology, active aortic signal monitoring, automatic respiratory signal extraction, and 3D radial acquisition with star-shaped K-space filling technology. This technique allows dynamic contrast-enhanced scanning during free breathing. To improve the comfort of elderly patients during examinations, and avoid examination failure due to the inability of elderly individuals to cooperate with breath-holding, this study compared the uCSR with that of the conventional breath-holding 3D-VIBE and evaluated whether the uCSR technique could replace the conventional breath-holding 3D-VIBE and serve as a routine liver dynamic contrast-enhanced scanning method for elderly patients with malignancy.

## Materials and methods

### General information

A prospective study was conducted between March 2023 and July 2024 and involved elderly patients aged ≥ 65 years who underwent liver MRI examinations. The inclusion criteria were as follows: (1) age of ≥ 65 years; (2) presence of malignancy (e.g., hepatocellular carcinoma, cholangiocarcinoma, ovarian cancer, cervical cancer, lung cancer, and other malignancies); (3) absence of contraindications or history of allergies to MRI contrast agents; (4) absence of renal function abnormalities; and (5) complete imaging data without metallic artifacts in the examination areas. The study included 100 cases: 59 males and 41 females. The patients’ age was 68.00 years (66.00–71.00). The participants provided informed consent, and the study was approved by the hospital’s Medical Ethics Committee (approval number: SCCHEC-02-2021-009).

## Instrumentation and methods

Scanning was performed using a United Imaging 3.0-T MRI scanner (UMR 780; United Imaging, Shanghai, China) equipped with an 18-channel phased-array body coil and a 32-channel spine coil. The patients were placed in the supine position with their heads first and their hands placed down on both sides of the body. A 6-h fast and water restriction period was required before the examination, and an indwelling needle was inserted into the elbow vein for contrast agent injection. Patients who underwent breath-holding liver dynamic contrast-enhanced examinations using the 3D-VIBE sequence were included in the 3D-VIBE group, whereas those who underwent free-breathing liver dynamic contrast-enhanced examinations using the uCSR technique were included in the uCSR group. Patients who failed to perform adequate breath-holding during T_1_-weighted imaging were automatically included in the uCSR group. Each group comprised 50 patients who were randomly allocated using the RANDBETWEEN (small value, large value) function of Excel, and the same scanning sequences were used for both groups, including consistent T_2_-weighted imaging (T_2_WI) in the coronal and transverse planes and 3D-VIBE imaging in the transverse plane. The 3D-VIBE group underwent three-phase scanning (i.e., arterial, portal venous, and delay phases), and the 3D-VIBE sequence was used during breath-holding. The uCSR group underwent uCSR sequence scanning during free breathing, and images for the arterial, portal venous, and delay phases were reconstructed. The scanning parameters of the T2-weighted imaging (T_2_WI), 3D-VIBE and uCSR sequence are shown in Table 1. The uCSR settings were as follows: pre-contrast phase 1 (temporal resolution for reconstruction, 45 s), arterial phase 2 (temporal resolution for reconstruction, 15 s), portal venous phase 1 (temporal resolution for reconstruction, 30 s), delay phase 1 (temporal resolution for reconstruction, 40 s), arterial input function, and region of interest (ROI) in manual mode (ROI diameter 10 mm). The 3D-VIBE sequence was performed with contrast injection triggered after 17 s for the arterial phase scan, 50 s for the portal phase scan, and 180 s for the delay phase scan. Then, 10 s after the start of the uCSR sequence, a high-pressure injector was used for the venous injection of the contrast agent (Gadopentetate Dimeglumine) at a speed of 2.0 mL/s. The contrast agent dose was calculated according to the patient’s weight (0.2 mL/kg). After the contrast agent was injected, 15 mL of saline was injected at the same rate as a saline flush.

**Table 1:** The scanning parameters of T_2_WI, 3D-VIBE and uCSR,

The scanning parameters	T_2_WI	3D-VIBE	uCSR
FOV	400 × 300 mm	400 × 280 mm	400 × 280 mm
TR	3001 ms	3.42 ms	3.08 ms
TE	82.68 ms	1.56 ms	1.4 ms
Slice thickness	5 mm	5 mm	5 mm
Slice gap	1 mm	None	None
Oversampling	20%	0%	0%
Matrix	256 × 230	320 × 288	256 × 256
Phase encoding	A > P	A > P	A > P
Flip angle	90°	10°	10°
Number of averages	1	1	1
Concatenations	2	1	1
Method for fat suppression	Chemical saturation	Chemical saturation	Chemical saturation
Compressed sensing acceleration factor	/	2.6	2
Number of spokes	/	/	3420
Phase cycling	/	/	Mode adaptive
Scan time	36 s	16 s	310 s

*FOV* field of view, *TR* repetition time, *TE* echo time.

## Image quality evaluation

### Qualitative analysis

All qualitative analyses were scored separately using a 5-point Likert scale. Two radiologists (radiologists 1 and 2, with 8 and 10 years of liver diagnosis experience, respectively) blindly evaluated the images using a subjective scoring system. The evaluation included two aspects: 1. Overall image quality of the 3D-VIBE and uCSR groups The scoring criteria were as follows: 5 = excellent, image quality with no artifacts, accurate phase timing, and suitable for diagnostic purposes; 4 = good, image quality with minimal artifacts, accurate phase timing and suitable for diagnostic purposes; 3 = moderate, image quality with some artifacts, partial phase timing inaccuracies, and mostly suitable for diagnostic purposes; 2 = poor, image quality with phase timing inaccuracies and some lesions not clearly visible, affecting diagnostic accuracy; 1 = very poor, image quality unsuitable for disease diagnosis. 2. Image artifacts (not limited to respiratory motion artifacts) in the 3D-VIBE and uCSR groups. The scoring criteria were as follows: 5 = excellent, no artifacts present; 4 = good, slight respiratory motion artifacts or slight radial artifacts; 3 = moderate, some respiratory motion artifacts or radial artifacts, generally suitable for diagnostic purposes; 2 = poor, significant artifacts affecting diagnosis; 1 = very poor, severe artifacts, unable to provide diagnostic recommendations.

### Quantitative analyses

Two experienced MRI technicians (technicians 1 and 2, with 7 and 10 years of experience, respectively) blindly measured the signal intensities of the liver parenchyma (SI_liver_) and erector spinae muscle (SI_muscle_) and the background signal standard deviation (SD_background_) on the same anatomical level in the three-phase images of both groups. The measurement levels for the signal intensity of the liver parenchyma and erector spinae were conducted at the level of the porta hepatis. The liver measurement area was confined to the right posterior hepatic parenchyma, whereas the erector spinae measurement area was restricted to the right erector spinae muscle tissue. The ROIs for each measurement had a diameter of < 0.5 cm. Three measurements were obtained and averaged, and the signal-to-noise ratio (SNR = SI_liver_ / SD_background_) and contrast-to-noise ratio (CNR = (SI_liver_ − SI_muscle_) / SD_background_) were calculated.

## Statistical analysis

Statistical analyses were performed using SPSSPRO (version 1.1.22) [6]. The Shapiro–Wilk test was used to evaluate the normality of the continuous variable distribution. Categorical or enumeration parameters are described as numbers (percentages), whereas continuous variables are expressed as the means ± standard deviations or the (medians (interquartile, ranges (IQRs)), as appropriate. The Mann–Whitney test was used for data that did not conform to the normal distribution, and the independent samples *t*-tests were used for data that conformed to the normal distribution. The intraclass correlation coefficient (ICC) was used to assess the inter- and intra-observer consistency of the data collected by the two radiologists and two technicians (< 0.2, poor consistency; 0.2–0.4, fair consistency; 0.4–0.6, moderate consistency; 0.6–0.8, good consistency; 0.8–1.0, excellent consistency). *p*-values < 0.05 were used to indicate statistical significance.

## Results

### Basic characteristics of the patients

Fifty-seven patients were initially included in the 3D-VIBE group; however, seven patients were excluded because of their inability to hold their breath. Approximately 87.7% of the patients in the 3D-VIBE group could perform breath-holding. The uCSR group comprised 50 patients, all of whom met the requirements and did not require breath-holding. The 3D-VIBE group had an average age of 68.00 years (66.00, 70.25), whereas the uCSR group had an average age of 67.00 years (66.00, 72.00). No statistically significant difference in age was found between the groups (*z* = −0.215, *p* = 0.830). The 3D-VIBE group comprised 19 females and 31 males, whereas the uCSR group comprised 22 females and 28 males (Table 2); however, no statistically significant difference in sex distribution was found between the groups (χ^2^ = 0.372, *p* = 0.542).

**Table 2:** The demographics of patients

Demographics		The 3D-VIBE group	The uCSR group
Age (years)		68.00 (66.00, 70.25)	67.00 (66.00, 72.00)
Sex (females/males)		19/31	22/28
Types of malignancy	Ovarian cancer	3	1
	Cervical cancer	2	4
	Hepatocellular carcinoma	7	4
	Lung cancer	8	10
	Rectal cancer	6	3
	Breast carcinoma	2	2
	Prostate cancer	5	2
	Nasopharyngeal carcinoma	3	4
	Glioma	0	1
	Gallbladder carcinoma	1	1
	Lymphoma	1	0
	Colon cancer	2	3
	Cholangiocarcinoma	1	3
	Malignant melanoma	1	0
	Renal cancer	1	3
	Pancreatic cancer	1	2
	Laryngeal cancer	1	0
	Bladder cancer	1	1
	Tongue cancer	0	1
	Thyroid cancer	0	1
	Carcinoma of penis	1	1
	Gastric cancer	4	2
	Laryngeal cancer	0	1

### Qualitative analysis

#### Overall image quality score

**Fig. 1:**
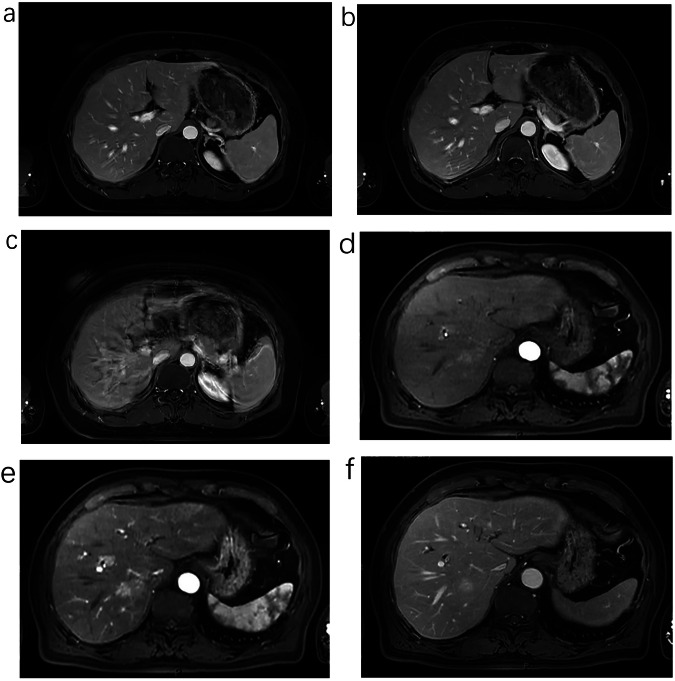
**a**–**c** Female, 65 years old, cervical cancer, liver dynamic contrast-enhanced scanning utilizes 3D-VIBE, Arterial Phase (**a**) is incorrect and slight breathing artifact, Portal Venous Phase (**b**) slight breathing artifact, and Delay Phase (**c**) significant respiratory artifact; overall image quality Score is 3. **d**–**f** Male, 68 years old, cholangiocarcinoma, liver dynamic contrast-enhanced scanning utilizes uCSR, Arterial Phase (**d**), Portal Venous Phase (**e**) and Delay Phase (**f**) have excellent image quality and accurate phase; the score is 5

The mean score for the 3D-VIBE group was 4.00 (4.00, 4.00), and the mean score for the uCSR group was 4.00 (4.00, 5.00). The results exhibited a statistically significant difference in the scores between the groups (*z* = 2.342, *p* = 0.019). This statistical result indicated that the overall image quality score was better in the uCSR group than in the 3D-VIBE group as shown in Fig. 1 and Table 3.

**Table 3:** Qualitative analysis

Group	Median (interquartile range)	Frequency					*z*	*p*-value
		1	2	3	4	5		
Overall image quality score (3D-VIBE)	4.00 (4.00, 4.00)	0	2	8	29	11	2.342	0.019
Overall image quality score (uCSR)	4.00 (4.00, 5.00)	0	0	2	30	18		
Image artifact score (3D-VIBE)	4.00 (4.00, 4.25)	0	1	10	27	12	2.105	0.035
Image artifact score (uCSR)	4.00 (4.00, 5.00)	0	0	2	31	17		

*3D-VIBE* the three-dimensional volume interpolated breath-hold examination, *uCSR* the united imaging compressed sensing with radial acquisition

#### Image artifact score

**Fig. 2:**
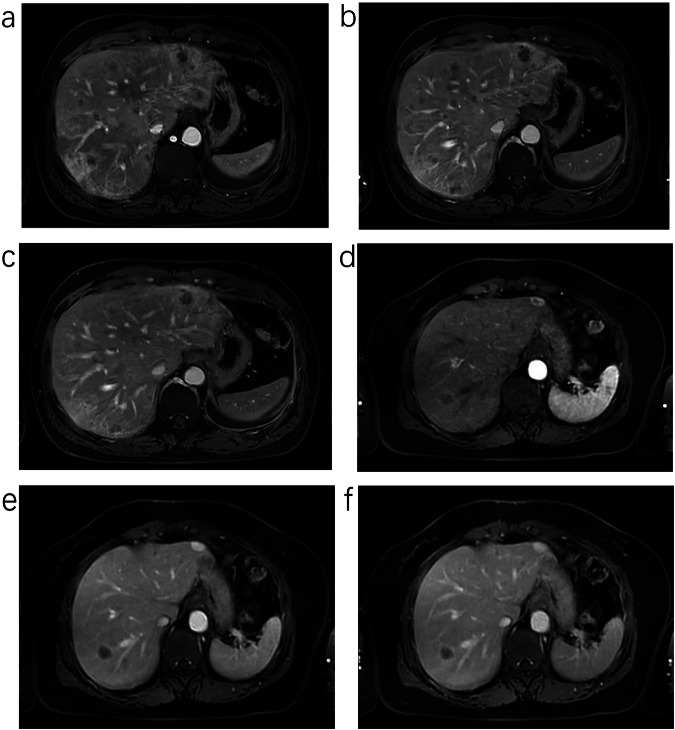
**a**–**c** Male, 66 years old, adenocarcinoma of the lung, liver dynamic contrast-enhanced scanning utilizes 3D-VIBE; client held his breath well; however, the boundary of blood vessels is blurred in the image, and some of them appear as bilateral shadows in Arterial Phase (**a**), Portal Venous Phase (**b**) and Delay Phase (**c**), the image artifact score is 3. **d**–**f** Female, 68 years old, hepatic metastases and hemangioma, liver dynamic contrast-enhanced scanning utilizes uCSR; there are no respiratory motion artifacts and other atypical motion artifacts in Arterial Phase (**d**), Portal Venous Phase (**e**) and Delay Phase (**f**), there are slight radial artifacts in Arterial Phase (**d**), the image artifact score is 4

The mean score for the 3D-VIBE group was 4.00 (4.00, 4.25), whereas that for the uCSR group was 4.00 (4.00, 5.00). The results exhibited a statistically significant difference in the scores between the two groups (*z* = 2.105, *p* = 0.035). This statistical result indicated that the uCSR group had better control over image artifacts than the 3D-VIBE group as shown in Fig. 2 and Table 3.

### Quantitative analyses

Arterial phase: The mean SNR and CNR values for the 3D-VIBE group were 388.40 (320.85, 424.08) and 150.10 (110.60, 192.20), respectively, whereas those for the uCSR group were 280.65 (234.64, 345.25) and 136.00 (113.60, 185.33), respectively. A statistically significant difference in SNR was found between the groups (*z* = 4.988, *p* = 0.000); however, no significant difference in CNR was observed (*z* = 0.676, *p* = 0.499). The results revealed that 3D-VIBE images had a higher SNR than uCSR images, and the CNR did not significantly differ between the groups (Table 4).

**Table 4:** Quantitative analyses

Group	Mean	*z/t*	*p*-value
The SNR of arterial phase (3D-VIBE)	388.40 (320.85, 424.08)	4.988	0.000
The SNR of arterial phase (uCSR)	280.65 (234.65, 345.25)		
The CNR of arterial phase (3D-VIBE)	150.10 (110.60, 192.20)	0.676	0.499
The CNR of arterial phase (uCSR)	136.00 (113.60, 185.33)		
The SNR of portal venous phase (3D-VIBE)	514.55 (426.78, 623.10)	5.674	0.000
The SNR of portal venous phase (uCSR)	368.80 (318.58, 415.95)		
The CNR of portal venous phase (3D-VIBE)	259.10 ± 84.269	3.638	0.000
The CNR of portal venous phase (uCSR)	205.89 ± 59.959		
The SNR of delay phase (3D-VIBE)	305.85 ± 57.248	5.471	0.000
The SNR of delay phase (uCSR)	240.54 ± 62.033		
The CNR of delay phase (3D-VIBE)	94.95 (70.90, 130.70)	1.258	0.208
The CNR of delay phase (uCSR)	84.85 (73.13, 104.88)		

*3D-VIBE* three-dimensional volume interpolated breath-hold examination, *uCSR* united imaging compressed sensing with radial acquisition, *SNR* signal-to-noise ratio, *CNR* contrast-to-noise ratio

Portal venous phase: The mean SNR and CNR values for the 3D-VIBE group were 514.55 (426.78, 623.10) and 259.10 ± 84.269, respectively, whereas those for the uCSR group were 368.80 (318.58, 415.95) and 205.89 ± 59.959, respectively. A statistically significant difference in SNR was observed between the groups (*z* = 5.674, *p* = 0.000); however, no significant difference in CNR was observed (*t* = 3.638, *p* = 0.000). The results observed that 3D-VIBE images had higher SNR and CNR values than uCSR images (Table 4).

Delay phase: The mean SNR and CNR values for the 3D-VIBE group were 305.85 ± 57.248 and 94.95 (70.90, 130.70), respectively, whereas those for the uCSR group were 240.54 ± 62.033 and 84.85 (73.13, 104.88), respectively. A statistically significant difference in SNR was found between the two groups (*t* = 5.471, *p* = 0.000); however, no significant difference in CNR was observed (*z* = 1.258, *p* = 0.208). The results revealed that 3D-VIBE images had a slightly higher SNR than uCSR images, and the CNR did not significantly differ between the groups (Table 4).

### Consistency analysis of subjective scores and objective measurements within and between the observer groups

Table 5 shows that the ICC values for all evaluation items within and between the observer groups were > 0.6 (intra-observer ICC: 0.891, 0.876, 0.904, and 0.943; inter-observer ICC: 0.838, 0.732, 0.867, and 0.908). The results revealed good data consistency within and between the observers.

**Table 5:** Intra- and inter-observer variability

	Intra-observer		Inter-observer	
	ICC	95% CI	ICC	95% CI
Overall image quality score	0.891	0.790–0.941	0.838	0.688–0.912
Image artifact score	0.876	0.763–0.933	0.732	0.493–0.857
SNR	0.904	0.818–0.949	0.867	0.746–0.928
CNR	0.943	0.905–0.973	0.908	0.831–0.925

*ICC* intraclass correlation coefficient, *CI* confidence interval, *SNR* signal-to-noise ratio, *CNR* contrast-to-noise ratio

## Discussion

In this study, 57 patients were recruited in the 3D-VIBE group; however, seven patients could not hold their breath. Therefore, 12.3% of patients could not use the traditional 3D-VIBE technique because they could not hold their breath. Nevertheless, using the uCSR technique, which does not require breath-holding, all cases were completed successfully. Therefore, the tolerance of patients to the uCSR was significantly higher than that to 3D-VIBE, and uCSR ensured the comfort of patients during examinations. The main reason is that uCSR enables 3D star-like radial acquisition in the K-space, which greatly reduces the influence of respiratory motion on images in contrast to linear Cartesian sampling used in 3D-VIBE [7, 8]. Furthermore, uCSR automatically extracts respiratory signals and generates a respiratory curve. The time during the free-breathing state, when the chest and abdomen are relatively motionless, is longer at the end of expiration than at the end of inspiration. Therefore, we used the bottom of the curve (end of expiration) as a reference and matched the acquired signals throughout the entire respiratory cycle to the respiratory curve. Different weights were applied to the data acquired from different respiratory curves during image reconstruction. The corresponding weight decreased as the data moved away from the bottom of the respiratory curve. This method ensures the use of acquired data while further reducing the impact of respiratory motion on image quality. However, some respiratory models cannot achieve high-quality images, which can be obtained only when breathing is consistent and uniform and the respiratory cycles have the same amplitude [9, 10]. When deep and shallow breaths alternate randomly, the mismatch between the top and bottom of the respiratory curve in the horizontal line results in noticeable radial artifacts in the reconstructed images. Therefore, patients should be instructed to maintain a consistent and stable breathing pattern before the examination.

In our study, two radiologists with expertise in medical image diagnosis subjectively scored the overall image quality and image artifact suppression ability of the two groups from a diagnostic perspective. The overall image quality score was slightly better in the uCSR group than in the 3D-VIBE group. When the seven excluded cases were included in the 3D-VIBE group, the advantage of the uCSR group over the 3D-VIBE group in subjective scoring was more pronounced. The uCSR can better address motion and cardiac pulsation artifacts than 3D-VIBE because radial acquisition has stronger motion robustness than linear Cartesian sampling [11].

In 3D-VIBE, respiratory motion occurring at any time during the entire scanning period will result in arc-shaped artifacts in all reconstructed images in the phase-encoding direction. For example, even if breath-holding is good for most scans, slight abdominal motion in a short period at the end can still cause motion artifacts, which are related to the magnitude of respiratory motion. However, upon detailed observation of the uCSR images in each phase, radiating streak artifacts appear at varying degrees in each phase instead of the typical arc-shaped motion artifacts caused by Cartesian line sampling, leading to a decrease in image quality. These radiating streak artifacts are mainly due to the use of radial K-space acquisition in uCSR [12].

The subjective scoring indicated that the tolerance of radiologists toward motion artifacts in 3D-VIBE was significantly lower than that toward streak artifacts caused by radial K-space acquisition in uCSR. The streak artifacts in uCSR can be improved to some extent by adjusting the sequence parameters, particularly by increasing the number of spokes or reducing the temporal resolution of the reconstructed data. However, when highly accurate arterial phase image timing is required, excessive reduction in temporal resolution may lead to nonstandard arterial phase reconstruction and potential contamination of portal venous phase acquisition data.

The timing window of the data required for image reconstruction is crucial for achieving satisfactory image quality in each phase, particularly in the arterial phase. Early or late data acquisition can result in premature or delayed arterial phase. In uCSR, the time–signal curve of a vessel is obtained by manually labeling the curve, and variations in the labeled arterial signal are considered during data acquisition. Then, based on the arterial time–signal curve as a reference, the time point at which the curve reaches half of its peak height is considered the start time. The start time determines the starting point of arterial phase reconstruction. By using this method, the reconstruction of each phase ensures the accuracy of the dynamic enhancement, particularly in the arterial phase of the liver. In this study, the abdominal aorta at the level of the porta hepatis was used as the labeled vessel, and the time–signal curve of the abdominal aorta was obtained. The multi-phase images reconstructed based on this curve effectively meet the diagnostic requirements.

Currently, a technique called XD-GRASP is widely used in research on the dynamic contrast-enhanced scans of the liver, uterus, rectum and other organs during free-breathing conditions [5, 13–15] and in adaptive radiation therapy for moving organs [16, 17]. This technique classifies the continuously acquired K-space data into multiple under-sampled datasets with different motion states. It uses motion signals directly extracted from the data for image reconstruction. Another study [6, 9, 18] used a novel Cartesian sampling technique called the XD-VIBE for dynamic contrast-enhanced scans of the liver. In the reconstruction of multi-phase images, the same temporal resolution is used to evenly divide the time points, and data from adjacent time points are reconstructed to produce multi-phase images. In this study, the image qualities of the arterial, portal venous, and delay phase images, including SNR and CNR, were objectively evaluated. No significant difference in CNR was observed between the arterial and delay phases. In terms of other comparison parameters, 3D-VIBE outperformed uCSR. The most important factor affecting the SNR and CNR in the reconstructed images obtained using uCSR is the temporal resolution of each phase. Decreasing the temporal resolution increases the amount of reconstructed data and significantly improves the SNR and CNR of the images. However, the accuracy of the phase-to-phase registration limits the reduction in the temporal resolution of image reconstruction, particularly in the arterial phase. The requirements for temporal resolution are relatively lower in the portal venous and delay phases than in the arterial phase, allowing an increase in the data volume by reducing the temporal resolution and thereby improving the image quality. For example, in this study, the temporal resolution of uCSR sequence parameters during the portal venous and delay phases was significantly lower than that during the arterial phase. In the future, we can also improve the SNR and CNR of the reconstructed image by modifying the parameters.

Currently, few studies have explored the clinical application of the uCSR technology in the free-breathing dynamic contrast-enhanced liver scans in elderly patients with cancer, uCSR and XD-GRASP combine compressed sensing [19] and 3D radial acquisition with star-shaped K-space filling technology [20]. The compressed sensing can improve the time resolution and also obtain satisfactory image quality [21], and the star-shaped K-space filling technology can significantly inhibit respiration-induced motion artifacts. According to subjective evaluations, uCSR performed better than 3D-VIBE. This finding may be attributable to suboptimal respiratory coordination in elderly patients with cancer and varying degrees of respiratory artifacts in the images. Furthermore, the accuracy of the arterial phase timing significantly influenced the subjective evaluation of the diagnosing physicians.

Although the SNR and CNR of uCSR images were lower than those in the 3D-VIBE group, they still maintained a good level, and uCSR had a higher subjective score, which also suggested doctors’ affirmation of the quality of uCSR images. Thus, we conclude that the importance of image artifacts and phase timing accuracy outweighed the importance of SNR and CNR in clinical diagnosis. Therefore, uCSR can be routinely used for dynamic contrast-enhanced liver scans in elderly patients with cancer because it can improve patient comfort and reduce the rate of failure due to inadequate breath-holding capability.

The limitations and shortcomings of this study include the small number of collected cases, which may have affected the statistical results. Future studies should aim to increase the number of cases to address this limitation.

## Supplementary information

Supplementary Data 1

## Data Availability

We have raw data and statistical results, and all relevant raw data can be queried from the hospital's PACS system.

## Abbreviations

3D-VIBE: Three-dimensional volume interpolated breath-hold examination
CNR: Contrast-to-noise ratio
ICC: Intraclass correlation coefficient
MRI: Magnetic resonance imaging
ROI: Region of interest
SNR: Signal-to-noise ratio
uCSR: United imaging compressed sensing with radial acquisition
